# Let us talk about mistakes

**DOI:** 10.1007/s00247-024-06034-z

**Published:** 2024-08-30

**Authors:** Erich Sorantin, Michael Georg Grasser, Ariane Hemmelmayr, Sarah Heinze

**Affiliations:** 1 Division of Pediatric Radiology, Department of Radiology, Medical University Graz, Auenbruggerplatz 34, 8036 Graz, Austria; 2 Diagnostic and Research Institute of Forensic Medicine, Medical University Graz, Neue Stiftingtalstrasse 6, 8010 Graz, Austria

**Keywords:** Cognitive psychology, Diagnostic errors, Equipment and supplies, Leadership, Radiology

## Abstract

Unfortunately, errors and mistakes are part of life. Errors and mistakes can harm patients and incur unplanned costs. Errors may arise from various sources, which may be classified as systematic, latent, or active. Intrinsic and extrinsic factors also contribute to incorrect decisions. In addition to cognitive biases, our personality, socialization, personal chronobiology, and way of thinking (heuristic versus analytical) are influencing factors. Factors such as overload from private situations, long commuting times, and the complex environment of information technology must also be considered. The objective of this paper is to define and classify errors and mistakes in radiology, to discuss the influencing factors, and to present strategies for prevention. Hierarchical responsibilities and team “well-being” are also discussed.

## Background

“Errare humanum est.” – a well-known statement by Cicero in his 12th “Philippica” addressing the Roman Senate [1]. As it seems, errors accompany us throughout our lives. Strategies to minimize errors have been developed in several areas of professional life, such as in the aviation industry.

In 1959, Garland was the first to evaluate diagnostic errors and discrepancies in radiology [2]. He conducted a study with 5,000 chest radiographs, which were rated by an expert panel. He found inter-individual and intra-individual discrepancy rates of 30% and 21%, respectively. Garland’s findings have been confirmed in subsequent studies – an overview was published by Berlin in 2007 [3].

In the USA, annual occurrence of medical errors or misinterpretations range from 44,000 to 400,000 cases, resulting in unplanned costs of about 17–29 billion dollars annually [4].

There are numerous reasons why errors can happen, ranging from incomplete/incompetent examinations (e.g., improper imaging protocol), technical errors (e.g., incorrect patient positioning, inappropriate filters) to diagnostic errors, missed findings, or misinterpretations. Image-guided therapy as an evolving part of radiology with its own inherent errors and procedure-related complications will not be discussed in this article as it focuses on diagnostic errors only.

Error rates depending on modality, as reported by Kim et al. [5], were most common in radiography (82%), followed by 30% in computed tomography (CT), 11.4% in magnetic resonance imaging, and 3% in bone scans. On the other hand, less than 1% were reported in fluoroscopy, ultrasound, and positron-emissions-tomography-CT (PET-CT). The most frequent errors in all modalities occurred in musculoskeletal imaging (66%) and body imaging (15%).

A radiology report, being the result of a high-level cognitive effort, must take into account multiple sources of information such as the patient’s history, the referring diagnosis, and the results of previous examinations. In addition, reporting takes place in the busy, complex environment of hospitals or radiology institutes/offices. The entire process is prone to errors. Being aware of typical errors and problems enables the radiologist to avoid them and develop appropriate strategies.

The objective of this article is to provide a definition and classification of diagnostic errors, related terms, and information on their frequency. The article also discusses contributing factors, both intrinsic and extrinsic, hierarchical responsibilities relevant for error prevention, suggestions for error management, and the potential role of artificial intelligence (AI).

## Definition of diagnostic errors

According to the National Academies of Sciences, Engineering, and Medicine (NASEM), a diagnostic error is defined as “a failure to either find an accurate and timely explanation of the patient’s health problem(s) or to communicate that explanation to the patient” [6]. Variations of this definition have been published, such as by Singh and Sitting’s “a missed opportunity to make a timely or correct diagnosis, or take the next diagnostic action step, based on available evidence at the time as a result of provider or system error” [7].

Discrepancies must be distinguished from medical errors. Discrepancies can be defined as “reasonable differences of opinion between radiologists about a finding or diagnosis”; however, in the worst case, both may be incorrect or incomplete [8].

## Classification of diagnostic errors and their frequencies

Kim et al. published an error classification scheme for radiological errors composed of 12 types, as shown in Table 1 [5]. A total of 656 cases with delayed diagnosis were analyzed by two radiologists, and error classification was achieved by consensus. The most common errors were under-reading (= missed findings), satisfaction with the search, faulty reasoning, and location (finding outside the area of interest).

**Table 1:** Classification scheme of radiological errors as derived from Kim et al. [5]

Type	Cause of error	Explanation	Frequency
1	Under reading	Findings were missed	82.0%
2	Satisfaction of search	The finding was overlooked due to the failure to pursue further anomalies after the initial anomaly being identified	44.0%
3	Faulty reasoning	Error of over-reading and misinterpretation occur when a finding is appreciated and interpreted as abnormal, but the cause is attributed incorrectly. In this category, we find misleading information and a limited differential diagnosis too	17.0%
4	Location	Finding was missed due to the positioning of the lesion outside the focal area of the image, such as in the periphery	14.0%
5	Satisfaction of report	Finding was missed due to a lack of attention to details in the report and an over-reliance on the radiology report from previous examinations	12.0%
6	Prior examination	Finding was missed due of failure to consult prior radiologic studies or reports	11.0%
7	Lack of knowledge	Finding is acknowledged but erroneously attributed to an incorrect cause due to a deficiency in the viewer’s or interpreter’s knowledge	6.0%
8	Technique	Finding was not identified due to the limitations of the examination or technique	4.0%
9	History	Finding is missed and overlooked due to the acquisition of an inaccurate or incomplete clinical history	3.0%
10	Complacency	Error of overreading and misinterpretation occur when a finding is appreciated but is attributed to the wrong cause	2.0%
11	Complication	Complication from a procedure	1.0%
12	Poor communication	Lesion is identified and interpreted correctly, but the message fails to reach the clinician	~ 0,0%

Sum is greater than 100.0% since some errors occurred in combination. Individual errors were ordered according to their frequency

**Fig. 1:**
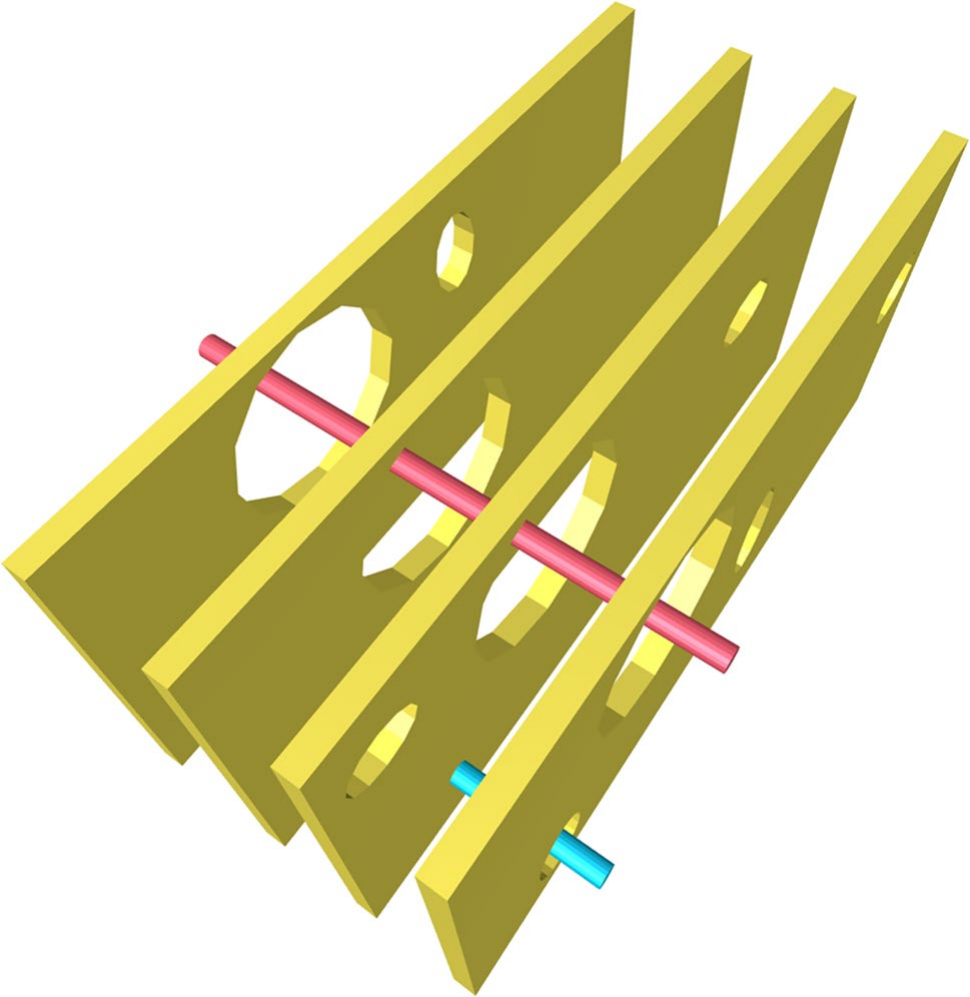
“Swiss Cheese Model” of errors (adapted from [9]). The *individual cheese slices* represent different layers of error prevention. *Holes* within the layers symbolize “latent” errors, which are blocked by the other error prevention layers – as symbolized by the *blue stick*. If error prevention layers are unluckily positioned, a latent error can lead to an active failure (*red stick*)

Brook et al. proposed a different classification scheme [9]. These authors distinguished between the following errors: (a) latent errors which are inherent to the system or the result of technical faults; (b) active errors which are the result of human error; (c) external causes are those that are beyond the control of the radiologist, including power failures and quenching; (d) patient-related causes like the failure of the patient to adhere to instructions or to demonstrate a lack of understanding of the procedures involved. Examples of active errors include diagnostic errors and misinterpretation, as well as complications from procedures. These can involve more than one person or result from latent errors. Additionally, they put forth the “Swiss Cheese Model,” which elucidates the manner in which latent errors may evolve into active errors (Fig. 1).

## Intrinsic factors

### Typology of man

C.G. Jung investigated the human typology and defined eight different archetypes (Table 2) [10, 11]. Myers and Briggs subsequently expanded upon these typologies, developing the Myers-Briggs type indicator (MBTI), which identifies six distinct personality types [12]. The “Big Five personality traits” model is currently the most widely accepted model of personality [13]—also known as the “OCEAN model.” The name of this model is an acronym for the first letters of the five factors defining it: openness, conscientiousness, extraversion, agreeableness, and neuroticism.

**Table 2:** Personality archetypes of C.G. Jung [10, 11]

Type	Explanation
Extraverted thinking	Principled, idealistic, objective, rational
Introverted thinking	Influenced by ideas, independent, often fearful of intimacy
Extraverted feeling	Adaptive, relating well to the external
Introverted feeling	Sympathetic, pleases others, may be dependent, reserved
Extraverted sensation	Realistic, concrete, pleasant, and friendly
Introverted sensation	Calm and passive, restrained, controlled, and controlling
Extraverted intuition	Enterprising, outgoing, can be irresponsible
Introverted intuition	Mystical, dreamer, and artist. Can be obsessive

Human personality is a complex and multidimensional phenomenon, and thus all models are simplifications. However, they provide an impression of how a person behaves, which is a valuable contribution to our understanding of the subject. It may be postulated that personality types exert an influence on the narrative of reports in radiology. Except for mammography, where the description of findings adheres to a strict, predetermined nomenclature, free text is employed for other imaging modalities and procedures.

Findings can be reported in a variety of ways, with the level of suspicion concerning the presence of a disease varying depending on the personality type of the individual radiologist reporting.

There are certain personality traits like perfectionism, high discipline, indecisiveness, idealism, high self-criticism, a high degree of empathy, and low flexibility, which increase the stress level of the person concerned and thus influence reporting [14].

### Chronobiology

Studies have revealed inter-individual variations in the circadian cycle, with age being a significant contributing factor [15, 16]. Failing to take account of the circadian cycle has been shown to have a detrimental impact on cognitive performance, learning, emotion regulation, and safety.

### Types of thinking

In 1974, Tversky and Kahneman described the basics of human decision making [4, 17]. Two distinct subtypes were identified, the heuristic (type I) and type II thinking. The heuristic approach to decision-making is a powerful method for making decisions based on limited information. It is also referred to as “intuitive thinking and acting.” This process is rapid, intuitive, and unconscious [4]. The type II thinking (system II) has been characterized as an analytical, slow, deliberate, and laborious process of decision-making [4]. In challenging circumstances, type II is the dominant process.

It is evident that type I is susceptible to bias, whereas type II promotes the likelihood of accurate diagnosis. The utilization of both types can fluctuate over time for a single individual, e.g., a radiologist at the onset of residency will be more likely to employ type II thinking when working through the corresponding checklist. As the residency progresses and the resident gains more experience and knowledge, thinking patterns shift towards type I.

### Cognitive bias

This can be defined as a “systematic pattern of deviation from norm or rationality in judgment,” with individuals creating their own “subjective reality” based on their perception of the input [18]. An alternative definition classifies cognitive bias as a “tendency to act in an irrational way due to our limited ability to process information objectively” [19].

To a certain extent, cognitive bias is an inherent aspect of human experience with our personal background, education, and experience, as well as other factors influencing our decision-making processes. In the field of radiology, cognitive bias represents a significant risk. The initial step in avoiding these cognitive biases is to gain an understanding of their nature. Table 3 provides a list of the most common types of cognitive bias and offers strategies for overcoming them [4, 19].

**Table 3:** Several types of cognitive bias and how to combat them (adapted from [4, 19])

Cognitive bias	Definition	How to combat
Alliterative bias (satisfaction of report)	Diagnosis influenced by a prior report—an incorrect impression was repeated	Make your own interpretation before reviewing others
Anchoring bias	Initial impression unduly influences further available/collected information	Access all available findings before making a diagnosis
Automation bias	Inappropriate reliance on computer-aided support over your own	Formulate your own impression before assessing computer-aided findings, and to be aware of the limitations of the software used
Availability bias	Unduly influence of experiences which can be recalled easily	Use information sources outside personal experience (guidelines, publications, colleague opinions)
Bandwagon effect (diagnosis momentum)	Mimicking thinking of (a maybe admired) another person	Do not dismiss your own opinion
Confirmation bias	Following an already predetermined diagnosis in your mind	Always carefully check all facts and prior findings. The alliterative bias falls in that category too
Framing bias	Influence how a referring diagnosis or clinical problem presented, e.g., question about a distinct diagnosis	Review images before reading referring diagnosis Make your mind free for considering an alternative diagnosis Afterwards check your findings in the light of the referring diagnosis
Hindsight bias (retrospective bias)	In retrospect everything is always clear	Take into consideration which information were available at the time of the study interpretation
Outcome bias	A tendency to favor a less severe diagnosis based on empathy for a patient	Check all facts and findings and be objective
Representativeness bias	Make a diagnosis of an image aspect based on your own perception	Rationalize your impression
Search satisfaction	“Radiologists are like dogs – they pick up one stick and miss the others”	Search all images with “restless eyes”
Zebra retreat bias	A rare (but correct) diagnosis will not be made due to lack of self confidence	If all requirements are fulfilled formulate the diagnosis
Attribution bias	Own findings influenced by patient characteristics	Check images first and review patient chart afterwards
Premature closure	A preliminary diagnosis is regarded as final	Formulate a working diagnosis and give a differential one
In-attentional bias	Findings which in unexpected locations or nature are missed	Always check the “big picture”

## Extrinsic factors

### Overstress

It is well documented that several stressors can have an adverse effect on performance, as outlined by Brown, Goske, and Johnson, and are listed in Table 4 [14].

**Table 4:** Common stress factors as published in [14]

Common stress factors for physicians	
Sitting for (failing) higher examinations	Increasing threats of litigation
Involvement in acute environments (Emergency Department, Neonatal Intensive Care Unit)	Decreasing cohesiveness (professional turf issues)
Juggling career/family	Decreased long-term unhurried relationships with patients
Overwork/tiredness	Increasing emphasis on “patients’ rights”
Non-English-speaking background	Involvement in medical catastrophe
Physical illness	Perceived decline in status of medicine
Authoritarian hierarchy’s intolerant of perceived “weakness of failure	Uncertainty about future career options
“System” issues (poor morale, shrinking funds)	Marital discord
Increasing emphasis on efficiency	Financial difficulties
Increasing requirements for formalized accountability	

A number of these factors can be summarized as a reduction in the environment appreciation, either within the department, from clinical colleagues, or in private life. It is evident that a combination of these factors will exacerbate the situation. Other factors such as sleep deprivation resulting from overnight shifts, parenthood, and career-related stress (e.g., board examinations, scientific work, job dependency on funds) are also contributing.

Other stressors in daily life include time-consuming commutes to work, delays in local transportation, and difficulties in finding parking. These factors contribute to punctuality issues and, consequently, elevated stress levels, particularly in the morning, causing a suboptimal start of the workday. Similarly, the rigid pick-up times for children at nurseries and other childcare facilities can be considered as a stressor too. It is thus evident that the often-cited concept of a “work-life balance” is not implemented at all.

### Information technology and legal aspects

What is more, in addition to the aforementioned factors, technical elements can also be a source of errors.

The transition from cathode-ray tube (CRT) monitors to liquid crystal display (LCD) monitors has been a notable advancement in the field of medical imaging. LCD monitors offer advantages over CRT monitors, including higher resolution, better contrast, and reduced space requirements [20].

The adoption of LCD monitors has rendered the maintenance of quality assurance a crucial undertaking. This necessitates that the monitors comply with the requisite standards for brightness, contrast, resolution, and color accuracy [20]. A failure to adhere to these standards may result in diagnostic errors or misinterpretations.

A typical monitor setup consists of a reporting workstation of monitors licensed for reporting purpose and one or more additional monitors, with lower specifications, typically used for word processing and database assessment [21].

Consequently, the radiologist is susceptible to inaccuracies when transferring imaging studies from the licensed monitors to the office ones, due to the lower geometric and radiometric resolution of the latter. Brook et al. emphasized the necessity of implementing a comprehensive system designed to identify and manage errors in medical imaging. This system should be capable of detecting diverse types of errors, including those that are latent and may not be immediately obvious, but which can still impact patient care [9].

It is of paramount importance for employers to select suitable equipment for medical imaging and for employees to utilize this equipment in an appropriate manner. Training and educating healthcare professionals is absolutely essential in order to guarantee their proficiency in effectively handling the equipment.

In Europe, this was addressed by directives based on the “European Union Medical Device Regulation 178/2002” [22]. Typically, these regulations must be implemented into national legislation – for example, in Germany, DIN 6868–157, and in Austria, ONR 195240–20 [22, 23].

**Fig. 2:**
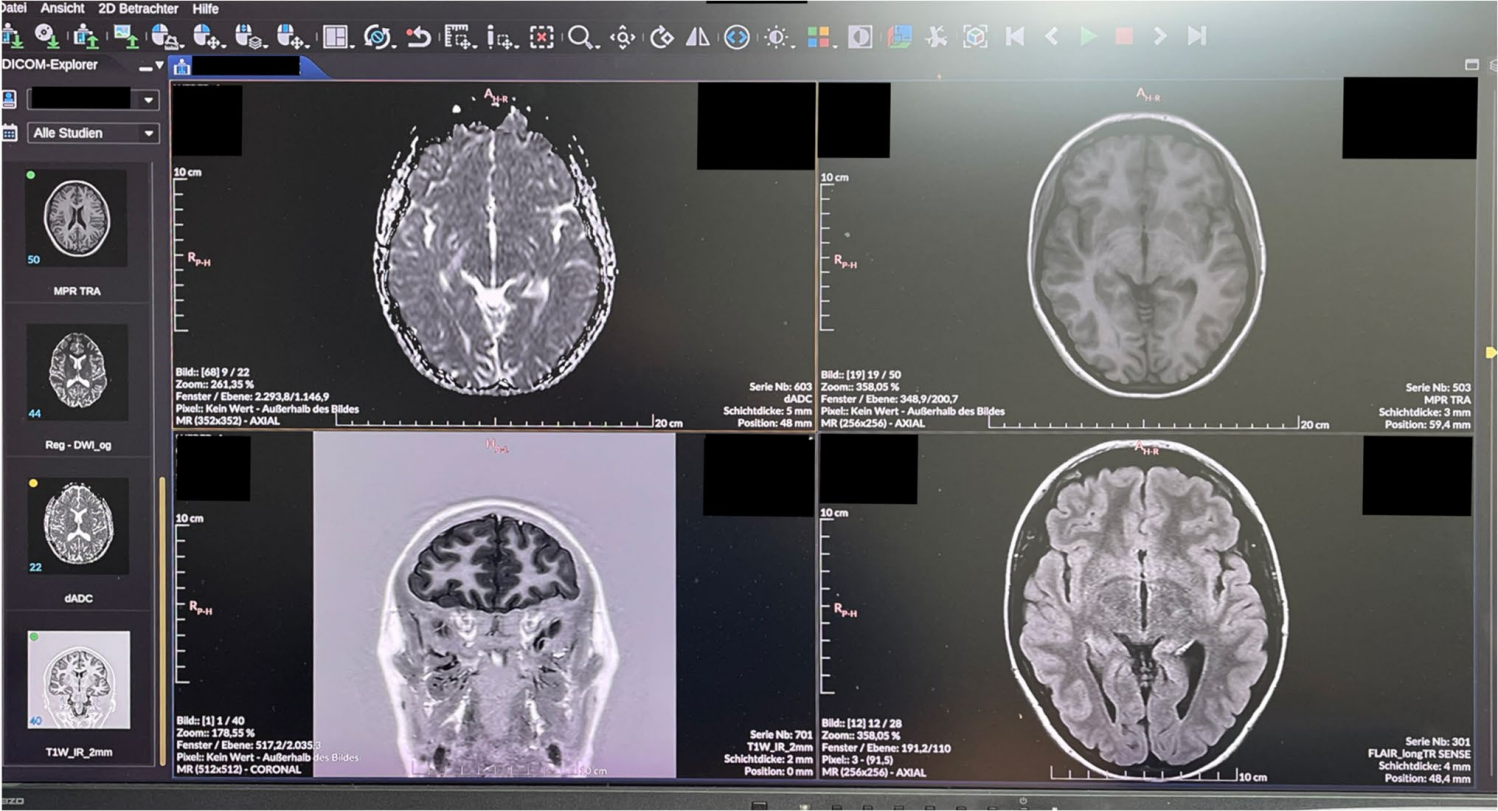
Photograph of a monitor oblique to two sunny windows. If the black background in the image in the right upper corner is compared to the background in the left upper corner, it can be recognized that the black background is gray instead of black – thus influencing diagnostic performance negatively due the contrast degradation

Both documents lay down the required maintenance and periodic quality control of medical monitors. The workstation setup must consider environmental factors such as ambient light. An example of an incorrect placement with monitor exposure to sunlight can be observed in Fig. 2. Modern devices are equipped with integrated front sensors that facilitate automated brightness calibration in accordance with the “Digital Information and Communications in Medicine (DICOM)” standard, thereby ensuring uniform image quality. As previously stated, incorrect placement of such systems biases the images, impairs their presentation, and consequently affects the quality of the radiological report.

It is also imperative to address the correct transfer of medical data. Emails containing data that can be traced back to an individual patient are not permitted. In Austria, the “Health Telematics Act 2012” establishes the regulatory framework for the secure transmission of medical data [24].

In order to guarantee the confidentiality of patients, the inclusion of notes regarding their medical conditions or diagnoses in public calendars is not in accordance with legislative requirements. The General Data Protection Regulation (GDPR) is directly applicable in the EU member states [25].

## Responsibilities

### Everybody

It is of the utmost importance that each individual ensures optimal functionality of their immediate work environment at all times. This encompasses a comprehensive examination of the workstation in use, ensuring that it is free from any technical issues, situated in an appropriate location, and equipped with the latest software and updated operating system. Additionally, it is essential to verify the availability of essential connections, such as archives and patient information systems. Other responsibilities include organizing a stress-free commute, having an escalation path in the event of sick children, and taking into account the stress factors already mentioned. It is, of course, essential to participate in the requisite of continuous medical education (CME) courses.

### Senior radiologist/consultant

It is incumbent upon the senior radiologist/consultant to ensure that examinations are conducted with the requisite quality and within the appropriate dose range. It is also incumbent upon the senior radiologist/consultant to report to the unit head any instances of frequent incorrect referrals, which may result in unnecessary exposure to radiation doses. A prompt review of the resident’s report is beneficial for all parties involved, as it helps to circumvent potential stressors.

### The department head/chair

It is the responsibility of the individual in this role to oversee all aspects of their respective unit. A significant objective is to enhance the team’s resilience. Resilience can be defined as the “ability to bounce back from adversity” [26].

In the field of medicine, it can be defined as the “capacity of individuals and groups to respond effectively to adversity” [27]. In the context of radiology, this can be understood as the capacity to adapt and overcome the challenges associated with patient care, technological advancements, increasing workloads, and evolving healthcare policies.

It is imperative that resilience is cultivated in order to maintain the highest standards of patient care, to mitigate the risk of burnout among team members, and to guarantee the long-term sustainability of radiology services.

Ensuring resilience is a multifaceted endeavor, with the establishment of a transparent organizational structure and the delineation of clear responsibilities representing two of the most crucial elements. A balanced workload is an effective method of preventing dissatisfaction within the team. Furthermore, active collaboration with related professional groups, such as radiographers and medical physicists, is a crucial aspect to consider. The current trend towards more part-time work provides the opportunity to better adapt schedules to individual needs of chronobiology.

Establishing a secure setting for the management of diagnostic errors, unidentified findings, and misinterpretation represents a further fundamental aspect of effective team leadership.

### Informational technology (IT) managers

IT managers need to ensure that the reporting devices and IT infrastructure comply with the relevant national and/or international legal requirements and function properly, including security updates.

### Error prevention and handling

In light of potential diagnostic errors, missed findings, or misinterpretations to occur, it is important that appropriate strategies are put in place, taking into account whether the issues arise within a department or unit, or between either.

#### Strategies within a department

The implementation of strategies within a department represents a multistage process. A review of residents’ reports is a mandatory requirement in many countries and is also a legal necessity. A culture of discussion and collaboration between colleagues and consultants should be fostered in order to address ambiguous findings. To avoid disrupting the workflow of others, it is recommended that two time slots per day be allocated for the discussion of controversial, non-urgent cases. The presence of radiologists on intensive care units facilitates the acquisition of more detailed clinical information, fosters a greater mutual understanding between clinicians and radiologists, and ultimately leads to more targeted imaging and the avoidance of unnecessary procedures in these vulnerable patients. Radiologists’ imaging visits have proven to be a valuable tool at the author’s institution.

A comprehensive library, radiologic textbooks, atlases, or digital versions are indispensable resources for quality management and education as are teaching files. Finally, “radiology discrepancy/errors” meetings are useful tools and have been further professionalized by the “Radiology Events and Learning Meeting” (REALM) approach [28]. In the Netherlands, for instance, these meetings are mandatory and subject to periodic review at 5-year intervals.

#### Strategies within the hospital

Interdisciplinary case discussions within hospital settings, involving all relevant clinical specialties, are a valuable tool for the acquisition of knowledge and the fostering of professional development. Furthermore, the implementation of an anonymous “Critical Incident Reporting System (CIRS)” has been demonstrated to be an effective strategy. The results from CIRS implementation in 22 regional, Austrian hospitals and a tertiary hospital have been reported by Sendlhofer et al. [29]. It is important to note that the majority of reported cases were associated with non-compliance with established guidelines.

In the event that a report is to be amended as a result of a second opinion or the introduction of new information (for example, a pathological report), it is imperative to retain the original report. A new report has to be created, incorporating the original one. At the end, a comment is made stating the rationale for the adaptions necessary. This is signed with the author’s name and date and followed by the new interpretation. Typically, hospital information systems incorporate a version control system for reports, with the most recent version always displayed on top. This allows the reader to trace the evolution of the report and understand the rationale behind the discrepancies between versions.

The hospital’s legal department should be involved in cases where a report has resulted in a treatment error of a patient or when forensic or legal implications are likely. In addition to any necessary action, the communication with the patient or provider should be discussed. Anecdotally, we find an offensive strategy in communicating with patients is a superior approach in contrast to remaining silent or attempting to downplay the problem.

### Potential role of artificial intelligence

The subject of new AI applications is currently the subject of intense discussion on a daily basis. AI has a long history [21], with the implementation of deep learning representing a significant development. In a statement made in 2016, Geoffrey Hinton, a prominent figure in the field of computer science and a key contributor to the development of deep learning technology, asserted that “It’s just completely obvious that within 5 years, deep learning is going to do better than radiologists” [30]. However, the current situation does not follow this prediction. An overview of the potential role of AI is provided by Gore et al., Choy et al., and Sorantin et al. [21, 31, 32].

“Large Language Models (LLM)” are AI applications that have been deployed as chat-bots on numerous commercial websites. The application of LLM to radiology has yielded varying degrees of success as shown in literature [33, 34].

What is more, the interactions between AI systems and radiologists are diverse, and the improvement in diagnostic performance is not always evident [35]. Thus, for the time being, it is difficult to predict whether these systems will prove effective in reducing errors and misinterpretations. Nevertheless, it is this author’s most profound conviction that AI applications such as image segmentation or a “personal digital assistant” will prove valuable to radiologists [21].

## Conclusion

To conclude, it is a given that humans are never free from mistakes and that a multitude of factors influence their decisions. These factors extend beyond the scope of education and encompass an individual’s personality, socialization, and numerous other elements. It is also essential to consider known stress factors and chronobiology. Consideration of the aforementioned factors is a first step towards prevention. It is vital that each individual assumes responsibility for their role within the hierarchy. To date, despite the multitude of documented and approved AI applications, their capacity to avert errors or misinterpretations remains indeterminate.
